# What Are the Costs of Diagnostics and Treatment of Lyme Borreliosis in Poland?

**DOI:** 10.3389/fpubh.2020.599239

**Published:** 2021-01-18

**Authors:** Anna Maria Rogalska, Olga Pawełczyk, Krzysztof Solarz, Tomasz Holecki

**Affiliations:** ^1^Department of Economics and Management in Health Care, Faculty of Public Health in Bytom, Silesian Medical University in Katowice, Bytom, Poland; ^2^Department of Parasitology, Faculty of Pharmaceutical Sciences in Sosnowiec, Medical University of Silesia, Katowice, Poland

**Keywords:** lyme borreliosis, diagnostics, treatment, health care cost, cost of illness

## Abstract

Lyme borreliosis (LB) is a growing epidemiological threat in many areas of the world, including North America and Europe. Due to the lack of effective protection against this disease, it seems important to ensure a timely diagnosis for effective treatment and the prevention of serious health consequences. The aim of this study was to assess the costs of diagnosis and treatment of Lyme disease in Poland. The costs incurred for the medical payer were analyzed. The cost of hospitalization due to LB for one patient in 2018 was estimated to be ~582.39 EUR, which constituted 53.10% of the average monthly salary of that year. In the analyzed period (2008–2018), the number of people treated by medical services due to Lyme disease increased, both in hospitalization and ambulatory specialist care. Although, the costs of hospitalization were the highest of the two, we noticed a change ratio between hospitalization and ambulatory specialist care in favor of the latter.

## Introduction

Lyme borreliosis (LB) is a multisystem infectious disease caused by bacteria from the *Borrelia burgdorferi* sensu lato complex. The main vectors of *B. burgdorferi* spirochetes in Europe are *Ixodes ricinus* ticks, belonging to the Ixodina suborder (1). Most human Lyme disease (LD) infections are caused by three spirochetal species: *B. burgdorferi* sensu stricto, *B. garinii*, and *B. afzelii* (2). This disease can manifest in many ways, because of the affinity of the spirochete for different tissues. The symptoms may include, among others, the occurrence of Erythema migrans (EM)–shortly after a tick bite in a feeding place, or acrodermatitis chronica athropicans (ACA)–a skin rash, which reveals itself many years after the infection. In addition, Lyme disease can affect the nervous system (neuroborreliosis), musculoskeletal system (Lyme arthritis), or heart (Lyme carditis), and if it left untreated, it could last for many months, even years, turning into a chronic form (3, 4). Because of the presence of many forms of Lyme disease, both diagnostics and treatment are challenging and can cause many difficulties (5).

The number of LB cases still increases, thereby the disease is classified as one of the most common vector-borne diseases in Europe, especially in Austria, the Czech Republic, Germany, Slovenia, and Switzerland. Additionally, a similar impact is noticed in places like China, Australia, Africa, and the north-east United States (6, 7). Every year, over 232,000 new cases of LD are reported in Europe (8). For example, the average annual morbidity over the last 10 years (2009–2018) in Bulgaria was 6.9/100,000 inhabitants (9), while in the Netherlands the total LB cases were estimated to be around 25,000 per year (145/100,000 inhabitants) (10). Infection rates are higher among children between the ages of 5 and 15 and for adults over the age of 50 (2).

Lyme disease is treatable in most cases, but its misdiagnosis or lack of treatment can result in devastating health consequences and may impose an excessive financial burden on healthcare (11). The estimated cost of treating acute and chronic Lyme disease in 2018 in the US was 4.8 billion (USD) and 9.6 billion (USD), respectively. In Europe, it was estimated at around 10.1 billion (EUR) for the acute form and 20.1 billion (EUR) for the chronic form (12).

The main purpose of this study was to examine the impact of LD on healthcare costs. The knowledge of medical services costs is important because it can contribute to an increase of awareness, and thus to the introduction of appropriate measures in health policy. In turn, the systematization of epidemiological data may be helpful in developing more accurate forecasts of Lyme disease risk. The comparison of the incidence of LB with the conducted health policy programs gives an overview of the areas where preventive programs were carried out and where they should be implemented in the future.

## Materials and Methods

### Study Design and Settings

The costs related to the diagnostics and treatment of Lyme disease were analyzed from the perspective of the medical services payer and from local government units (costs of health policy programs regarding diagnostic tests of Lyme disease). All cost values were standardized into EUR and the data of average salary in a given year came from the National Bank of Poland.

### Data Collection

Data on the costs of health services obtained from the benefited payer were extracted on the basis of the International Statistical Classification of Diseases and Health Problems–Tenth Revision (ICD-10) code: A69.2 “Lyme disease. Chronic erythema migrans caused by *B. burgdorferi* sensu lato. Tick-borne spirochetosis. Lyme borreliosis.” The analysis of LB costs was based on a retrospective assessment of health insurance data for the years 2008–2018 which were provided by one of the paying wards. Information regarding both the costs of hospitalization and ambulatory specialist care services came from the Silesian Voivodeship, the second largest in Poland with almost five million inhabitants. According to the territorial classification of the European Union, this area is classified as NUTS 2 (Nomenclature of Territorial Units for Statistics), which is also comparable in terms of tasks, size, and structure to British counties and German federal states.

### Calculations

To calculate the costs of antibiotic therapy, a monthly period of the administration of the three most frequently recommended active substances in the treatment of LD was adopted, including: amoxicillin, doxycycline, and cefuroxime which are available on the Polish market. A total of 100% of the drug price (no reimbursement) from 10 pharmacies was taken into account and the minimum and maximum value were calculated. Two strategies were adopted for the calculations: (a) when erythema migrans is presented–the antibiotic therapy was recommended; (b) when suggestive manifestations of the infection caused by *B. burgdorferi* (i.e., Borrelial lymphocytoma BL, Acrodermatitis chronica atrophicans ACA, Lyme arthritis, Lyme carditis, or neuroborreliosis) are presented - the two-stage serological diagnostics were recommended. Enzyme immunoassays (EIA) were used as screening tests for the detection (depending on the clinical form of LB) of IgM or IgG antibodies specific for *B. burgdorferi* in the serum. If the sample result was positive or equivocal after EIA, an additional analysis was performed using Western-Blot antibody determination (Table 1). A positive serological result, without any clinical symptoms for LB did not allow for the start of proper treatment (13, 14).

**Table 1:** Estimated costs of treatment of Lyme disease in Poland.

**Types of diagnostics**	Way of treatment^**^	Minimum amount^*^	Maximum amount^*^
Erythema migrans + medical visit	Antibiotic therapy	13.40	30.47
Clinical symptoms + medical visit + enzyme immunoassay (EIA) + WESTERN-BLOT	Antibiotic therapy	119.32^***^	136.15^***^
Clinical symptoms + medical visit + PCR reaction (in special cases)	Antibiotic therapy	Not estimated	Not estimated

* *Values given in EUR per patient*.

** *Drug prices are included without refunds*.

*** *EIA costs in health policy programs (reports)*.

In order to calculate an average cost of the ambulatory specialist care and hospitalization of LB, the cost of settled medical procedures in a given year were divided by the number of hospitalized patients in a given year. To make Lyme disease treatment expenses more realistic, they were compared with the average monthly salary in Poland (Tables 2, 3).

**Table 2:** Hospitalization costs for ICD-10 A 69.2 in 2008–2018 in the Silesian Voivodeship in Poland.

**Year**	**Number of people hospitalized for ICD-10 A69.2**	**Number of health services provided in a given year**	**Average number of services per patient**	**Value of all health services in EUR**	**Value of health services per patient in EUR**	**Average value of earnings in a given month in EUR**
2008	366	410	1.12	153490.29	419.37	857.17
2009	1,215	1,439	1.18	458233.45	377.15	722.08
2010	1,011	1,101	1.09	579666.42	573.36	812.10
2011	1,219	1,322	1.08	702981.02	395.12	847.64
2012	1,029	1,150	1.12	590814.11	574.16	857.86
2013	1,176	1,271	1.08	646143.57	400.79	882.54
2014	1,316	1,431	1.09	719268.18	546.56	918.61
2015	949	1,027	1.08	517678.95	545.50	949.28
2016	1,277	1,396	1.09	703378.67	550.81	944.64
2017	1,206	1,239	1.03	639277.57	530.08	1020.38
2018	1,253	1,335	1.07	729743.47	582.40	1096.81
Average	1092.45	1192.82	1.09	585515.97	499.57	–

**Table 3:** Costs of ambulatory specialist care (ASC) health services provided in 2008–2018 due to ICD-10 A 69.2 in the Silesian Voivodeship in Poland.

**Year**	**Number of people using ASC**	**Number of health services that year**	**Average number of services per patient**	**Value of all health services in EUR**	**Value of all health services per patient in EUR**	**Average value of earnings in a given year in EUR**
2008	1,872	2,190	1.17	25794.57	13.78	857.17
2009	3,243	4,782	1.47	51967.99	16.02	722.08
2010	3,768	5,890	1.56	72596.67	19.27	812.10
2011	7,055	8,043	1.14	135959.42	19.27	847.64
2012	8,233	9,899	1.20	209838.22	25.49	857.86
2013	8,770	11,027	1.26	235605.51	26.86	882.54
2014	10,806	13,089	1.21	292310.99	27.05	918.61
2015	10,290	12,470	1.21	311227.90	30.25	949.28
2016	11,129	12,957	1.16	305309.17	27.43	944.64
2017	12,809	13,512	1.05	269147.50	21.01	1020.38
2018	13,311	15,252	1.15	361941.07	27.19	1096.81
Average	8,299	9919.18	1.23	206518.09	23.06	–

In Table 2, we present the sum of all medical services performed during hospitalization in a given year, as well as the number of patients who were hospitalized for Lyme disease. It showed an average number of medical services per patient during hospitalization. We made analogous calculations for ambulatory specialist care (Table 3).

In order to estimate the cost of LB diagnostics and treatment, reports from the implemented health policy programs regarding ICD-10 A69.2 were used. These programs were carried out by local government units and assessed by the Agency for Health Technology Assessment and Tariffs in 2010–2018. The list of reviewed programs included 12 decisions regarding the Lyme disease health policy program, of which eight were implemented as of 2018. The analysis included programs financed from the city or county budget. These programs were carried out in three out of 16 voivodeships: Silesian, Pomeranian, and Greater Poland.

## Results

### Costs of Therapy With Antibiotics

The first analysis showed the range (maximum to minimum) of medical costs caused by the treatment of Lyme disease per patient in Poland (Table 1). The monthly cost of antibiotic treatment in 2020 ranged from 13.40 to 30.47 EUR in Poland, excluding drug reimbursement. The cost of the treatment was passed onto the patient (not including reimbursement) if one of the three recommended antibiotics were used.

### Hospitalization Costs

The analysis showed that the number of people hospitalized due to ICD-10 A69.2 has been steadily increasing over the studied 10 years and ranged from min = 366 in 2008 to max = 1316 in 2014. Hospitalization costs per patient in 2008 amounted to 419.37 EUR (which accounted for 48.92% of the average monthly salary) and in 2018 amounted to 582.39 EUR, which constituted 53.10% of the average monthly salary that year. In turn, the average number of medical services per patient was 1.09 (min = 1.08; max = 1.12) (Table 2). It means, that approximately one patient benefited from one medical service during hospitalization.

### Costs of Ambulatory Specialist Care (ASC)

As a rule, the value of ambulatory specialist care is much lower than the cost of hospitalization, but on the other hand, a much larger group of patients uses this kind of medical services. Ambulatory specialist care due to Lyme disease is constantly evolving. In 2008, 1,872 patients were treated with ASC, and in 2018–13,311 (an increase by 611%). The average number of benefits in ASC per capita was = 1.23 (min=1.05; max=1.56). The costs of ambulatory specialist care in 2008 per patient amounted to 13.78 EUR (which was 1.60% of the average salary in Poland that year), and in 2018 they amounted to 27.19 EUR (which was 2.48% of the average monthly salary in Poland that year) (Table 3).

### Share of Hospitalization Costs in Total Costs

**Figure 1:**
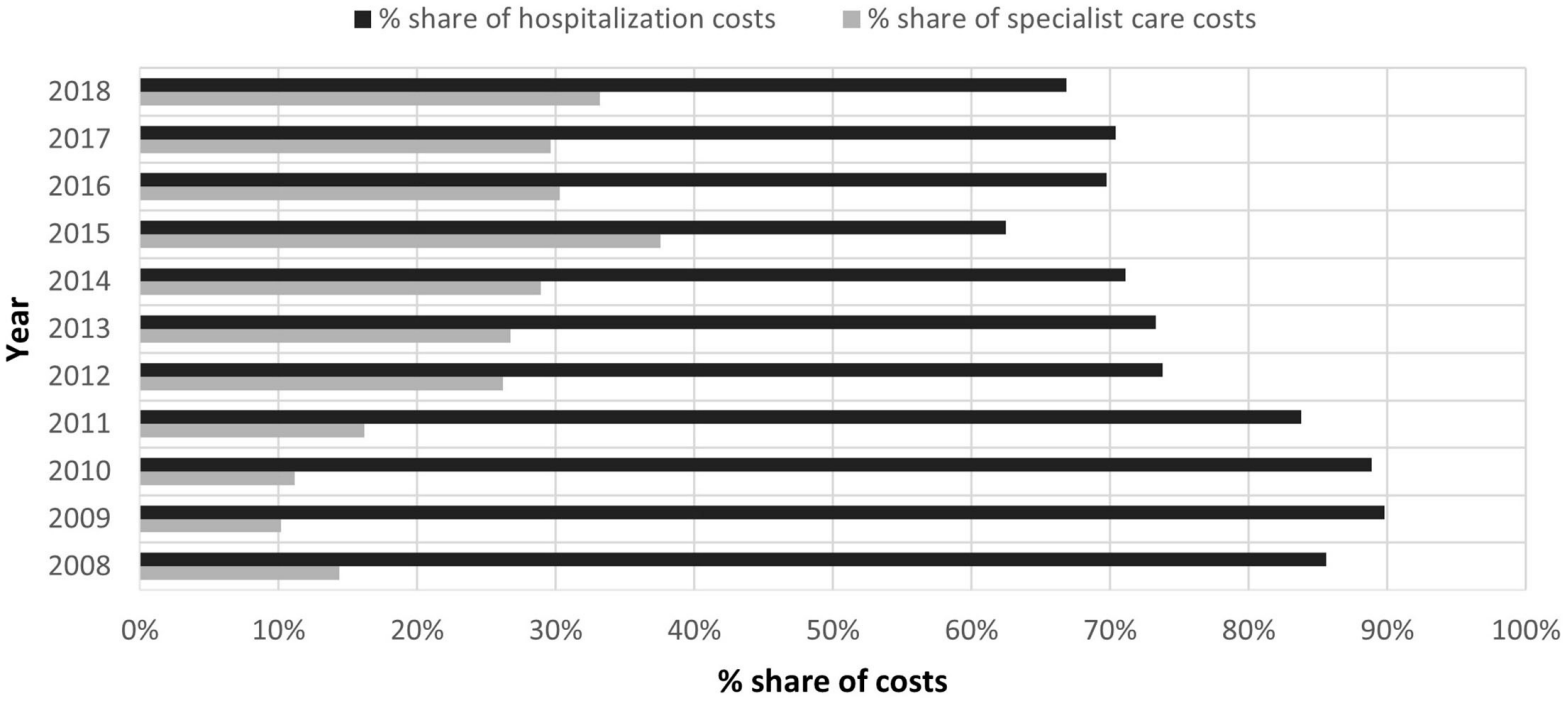
Comparison of the percentage share of ambulatory specialist care costs (ASC) and the costs of hospitalization to the total expenses of the payer of services due to ICD-10 A 69.2 in 2008–2018.

The share of hospitalization costs in 2009 was the highest and accounted for 90% of the total costs. However, over time, it began to decrease and in 2015 it reached the lowest value (taking into account the analyzed period of 2008–2018), i.e., 62%. In 2018, it reached 67% of the total costs (Figure 1).

### Costs of the Health Policy Program

Due to the increasing number of Lyme disease incidence in Poland over consecutive years, some local government units have introduced health policy programs for LB. They were intended to examine a part of the population living in a specific area in order to diagnose or rule out the occurrence of this disease earlier. By far the highest incidence of Lyme disease has occurred for several years in eastern Poland–in the Podlaskie Voivodeship (15). The highest level was reached in 2016–134.9/10,000 inhabitants, which is a value significantly exceeding the average for the whole country, and this area has also been in first place in terms of incidence in relation to the entire country invariably since 2010 (Table 4).

**Table 4:** Incidence of Lyme disease per 100,000 inhabitants in the voivodeships of Poland (2010–2018).

**Voivodeships/Year**	**2018**	**2017**	**2016**	**2015**	**2014**	**2013**	**2012**	**2011**	**2010**
Lower Silesian	29.3	29.4	32.1	19.1	17.8	20.1	16.2	22.6	19.4
Kuyavian-Pomeranian	17.7	26.2	29.0	19.1	19.8	20.5	16.6	15.6	17.2
Lublin	92.0	92.8	89.2	51.0	39.7	37.8	30.4	39.0	34.3
Lubusz	60.6	78.5	63.5	40.8	37.0	34.2	27.2	28.7	35.3
Łódz	24.2	25.7	32.2	17.8	16.1	11.6	8.7	9.0	8.4
Lesser Poland	107.2	107.2	87.3	51.2	53.4	54.1	24.8	21.7	23.2
Masovian	30.0	41.2	40.8	26.8	27.1	27.7	14.2	15.9	17.2
Opole	70.2	65.0	68.2	39.4	41.7	42.8	34.8	33.3	29.2
Subcarpathian	81.8	69.6	51.6	37.2	47.9	46.4	31.6	33.4	32.0
Podlaskie	108.8	130.1	134.9	96.3	106.8	100.2	81.4	75.7	76.0
Pomeranian	49.1	63.2	56.9	38.4	31.4	22.1	13.2	9.1	6.2
Silesian	57.8	61.0	71.5	45.9	57.1	49.5	35.4	37.2	32.8
Holy Cross	33.8	36.0	31.7	20.2	21.8	14.9	8.5	12.1	14.1
Warmian-Masurian	91.2	90.7	97.2	75.1	62.5	50.8	47.7	53.8	61.9
Greater Poland	14.9	18.8	16.6	11.0	9.0	7.0	6.2	5.3	7.5
West Pomeranian	45.1	49.9	49.7	30.3	29.9	25.9	15.4	14.0	15.1

*NIZP-PZH (www.pzh.gov.pl)*.

The Lublin Voivodeship has been excluded from the analysis due to the ongoing implementation of the several-year Lyme disease program financed from European funds. Health policy programs concerning the diagnosis of Lyme disease were implemented in communes of voivodeships with an average incidence in the country. In the Silesian Voivodeship, health policy programs were implemented to the highest degree–in five communes/counties. In turn, in the Greater Poland and Pomeranian Voivodeships, they implemented one program per commune or county. The total average cost of the health policy program was 16996.93 EUR (min = 3200.23 EUR, max = 64391.10 EUR, where EUR = 4.27 PLN–EUR exchange rate in 2018), which constituted 129.14% of the average annual salary in Poland. For some areas, health policy programs have been prepared and positively assessed by the Agency for Health Technology Assessment and Tariffs, but ultimately not implemented due to the lack of financial resources in a given local government unit (Source: reports from the implemented health policy programs).

## Discussion

This study belongs to a small group of reports that shows the economic burden of Lyme disease on the Polish healthcare system. Estimating the medical costs of LD is extremely difficult due to a number of factors, which include: the time frame and used methods of diagnosis (an acute or chronic form of the disease), the severity of the disease, the symptoms, and treatments associated with it (16).

The diagnosis of borreliosis at the early stage is extremely important, and difficult, if there were no characteristic symptoms after a tick bite and it was not registered by the patient. According to the guidelines of the Polish Society of Epidemiologists and Medical Doctors of Infectious Diseases, the treatment of Lyme disease at the early stage of the disease varies, depending on the presence or absence of erythema migrans, applied diagnostics, and antibiotic therapy. This has a significant impact on the cost of treatment (17).

In a study performed by Maes et al. describing an analysis model for borreliosis treatment decisions, they presented the costs of a medical visits, diagnostic tests, and antibiotic therapy in the case of acute Lyme disease (18). In that study, the estimated cost was > 100 USD, which is a similar result (averaging the currency of 100 USD = EUR 90) to our study. The two-stage diagnostics tests combined with antibiotic therapy costs were between 119.32 and 136.15 EUR. Another study by Davidsson (12) presented the costs of antibiotic therapy for Lyme disease per month in Europe. The value was 90,589,198 EUR with an infection number of 2,448,357, which amounts to ~37 EUR per person. This amount is slightly higher, than in our study, where the costs of monthly antibiotic therapy without reimbursement were 13.40–30.47 EUR and depended on the type of applied substances.

Our analyzes showed the average number of people hospitalized in Silesia, Poland, due to Lyme disease in the years 2008–2018, which was 1,093 people per year. The peak of the LD incidence was in 2013, when 1,316 patients were hospitalized. However, this number is lower than in a Germany based on a study performed by Lohr et al. (19), where an average of 7,500 people were admitted for hospital treatment (2008: *n* = 8009; 2009: *n* = 6849; 2010: *n* = 7626; 2011: *n* = 7505). In our study, the hospital costs from the perspective of the payer of the services in 2018 were estimated at 7.29 million EUR, which is significantly less than in the research from Germany, where the costs were about 23.7 million EUR per year (19). In the study from the United States, the mean cost of one hospitalization due to Lyme borreliosis was 15,683 USD (20). In comparison to our study, where the average cost of hospitalization per patient was 499.57 EUR (15,683 USD = 13,212.90 EUR), the costs intended for hospitalization in the United States were much higher.

According to data from research conducted in the United States between 2006 and 2010, Lyme disease was associated with a higher total healthcare cost by 2,968 USD in comparison to the cost for patients without LB. More precisely, the cost increase was estimated by 464 USD for outpatient evaluation and treatment, and by 612 USD on medicines over a 12-month period (21). Compared to the above study, the costs of LD healthcare in Poland in 2018 were lower and amounted to 421.09 EUR (31.27 EUR per patient). These differences are probably related to a different methodology and the lack of consideration of social costs in this study.

In order to reduce the costs connected with Lyme disease diagnostics and treatment, the most important task is to raise public awareness about preventive measures that help to protect against tick bites. As public awareness rises, healthcare professionals will be able to intervene earlier when EM or alarming symptoms appear after a tick bite, which means that both social and economic costs of this disease could be reduced. It should be emphasized that the use of antibiotic therapy at the initial stage of the disease is highly effective during treatment, does not require hospitalization, and thus does not incur high costs, both for the patient and the healthcare system.

In conclusion, the cost of diagnostics and treatment of Lyme disease in this study was dependent on the stage of the disease. It can be seen that for people diagnosed quickly with EM, the costs of treatment were the lowest. In turn, for people requiring hospitalization, the cost was the highest. Our study presented a relatively low impact on financial resources for hospital treatment of Lyme disease compared to the results of other researchers. However, the cost of hospital treatment accounted for about a half of the average monthly salary in Poland during the analyzed period. Between 2008 and 2018, the number of hospitalizations for ASC services gradually decreased, which is a positive trend because they are less expensive.

## Data Availability Statement

The original contributions presented in the study are included in the article/supplementary materials, further inquiries can be directed to the corresponding author/s.

## Author Contributions

AR had an idea. AR, OP, and KS contributed to the writing of the text. TH carried out a final revision. All authors contributed to the article and approved the submitted version.

## Conflict of Interest

The authors declare that the research was conducted in the absence of any commercial or financial relationships that could be construed as a potential conflict of interest.

## References

[1] Stanek G Wormser GP Gray J Strle F Lyme borreliosis. Lancet. (2012) 379:461–73. 10.1016/S0140-6736(11)60103-7 21903253

[2] Mead PS. Epidemiology of lyme disease. Infect Dis Clin North Am. (2015) 29:187–210. 10.1016/j.idc.2015.02.010 25999219

[3] Geebelen L Van Cauteren D Devleesschauwer B Moreels S Tersago K Van Oyen H. Combining primary care surveillance and a meta-analysis to estimate the incidence of the clinical manifestations of Lyme borreliosis in Belgium, 2015-2017. Ticks Tick Borne Dis. (2019) 10:598–605. 10.1016/j.ttbdis.2018.12.007 30772196

[4] Steere AC Strle F Wormser GP Hu LT Branda JA Hovius JW Lyme borreliosis. Nat Rev Dis Prim. (2016) 2:16090 10.1038/nrdp.2016.90 27976670 PMC5539539

[5] Smith AJ Oertle J Prato D Chronic lyme disease: persistent clinical symptoms related to immune evasion, antibiotic resistance and various defense mechanisms of borrelia burgdorferi. Open J Med Microbiol. (2014) 4:252–60. 10.4236/ojmm.2014.44029

[6] Luché-Thayer J Updating ICD11 Borreliosis Diagnostic Codes. Edition One March 29, 2017. Glob. Netw. Institutional Discrim. Ad Hoc Comm. Heal. Equity ICD11 Borreliosis Codes. (2017).

[7] European Centre for Disease European Centre for Disease Prevention and Control. An agency of the European Union: Tick species - Distribution maps (2020). Available online ar: https://www.ecdc.europa.eu/en/disease-vectors/surveillance-and-disease-data/tick-maps (accessed November 10, 2019).

[8] Sykes RA Makiello P. An estimate of Lyme borreliosis incidence in Western Europe. J Public Heal. (2017) 39:74–81. 10.1093/pubmed/fdw017 26966194

[9] Ermenlieva N Tsankova G Todorova TT. Epidemiological study of Lyme disease in Bulgaria. Cent Eur J Public Health. (2019) 27:235–8. 10.21101/cejph.a5007 31580560

[10] Hofhuis A Harms M Bennema S Van Den Wijngaard CC Van Pelt W Physician reported incidence of early and late Lyme borreliosis. Parasites Vectors. (2015) 8:161 10.1186/s13071-015-0777-6 25889086 PMC4363353

[11] Mac S da Silva SR Sander B. The economic burden of lyme disease and the cost-effectiveness of lyme disease interventions: a scoping review. PLoS ONE. (2019) 14:e0210280. 10.1371/journal.pone.0210280 30608986 PMC6319811

[12] Davidsson M. The financial implications of a well-hidden and ignored chronic lyme disease. Pandemic Healthcare. (2018) 6:16. 10.3390/healthcare6010016 29438352 PMC5872223

[13] Flisiak R Pancewicz S. Diagnostics and treatment of Lyme borreliosis. Recommendations of polish society of epidemiology and infectious diseases. Przegl Epidemiol. (2008) 62:193–9. 18536243

[14] Pancewicz SA Garlicki AM Moniuszko-Malinowska A Zajkowska J Kondrusik M Grygorczuk S. Diagnosis and treatment of tick-borne diseases recommendations of the polish society of epidemiology and infectious diseases. Przegl Epidemiol. (2015) 69:309–16. 26233093

[15] Rosińska M Infectious diseases and poisonings in Poland in 2010-2018 Chief Sanitary Inspectorate – Department for Communicable Disease and Infection Prevention and Control. National Institute of Hygiene – Department of Epidemiology (2019). 73, 4 Available online at: http://www.przeglepidemiol.pzh.gov.pl/files/peissues/PE_4_2019__SRODEK_z_OKLADKA.pdf (accessed November 10, 2019).

[16] Joss AW Davidson M Ho-Yen DO Ludbrook A. Lyme disease—what is the cost for Scotland? Public Health. (2003) 117:264–73. 10.1016/S0033-3506(03)00067-2 12966749

[17] Zhang X Meltzer MI Peña CA Hopkins AB Wroth L Fix AD. Economic impact of Lyme disease. Emerg Infect Dis. (2006) 12:653–60. 10.3201/eid1204.050602 16704815 PMC3294685

[18] Maes E Lecomte P Ray N. A cost-of-illness study of lyme disease in the United States. Clin Ther. (1998) 20:993–1008. 10.1016/S0149-2918(98)80081-7 9829450

[19] Lohr B Müller I Mai M Norris DE Schöffski O Hunfeld KP. Epidemiology and cost of hospital care for Lyme borreliosis in Germany: lessons from a health care utilization database analysis. Ticks Tick Borne Dis. (2015) 6:56–62. 10.1016/j.ttbdis.2014.09.004 25448420

[20] Schwartz AM Shankar MB Kugeler KJ Max RJ Hinckley AF Meltzer MI. Epidemiology and cost of Lyme disease-related hospitalizations among patients with employer-sponsored health insurance-United States, 2005-2014. Zoonoses Public Health. (2020) 67:407–15. 10.1111/zph.12699 32462811 PMC7521202

[21] Adrion ER Aucott J Lemke KW Weiner JP. Health care costs, utilization and patterns of care following lyme disease. PLoS ONE. (2015) 10:e0116767. 10.1371/journal.pone.0116767 25650808 PMC4317177

